# Supplementary material for: Modeling glioblastoma heterogeneity as a dynamic network of cell states
Source: Mol Syst Biol. 2021 Sep 16;17(9):e10105. doi: 10.15252/msb.202010105 (PMC8444284; doi:10.15252/msb.202010105)
Supplement: Supplementary file 6 — Source Data for Figure 5 [file MSB-17-e10105-s004.zip › Figure5A_sourcedata/GSEA_3071/hallmarks_stateVI.GseaPreranked.1623742557031/HALLMARK_MITOTIC_SPINDLE.html]

Details for gene set HALLMARK\_MITOTIC\_SPINDLE[GSEA]

|  || Dataset | state63071 |
| Phenotype | NoPhenotypeAvailable |
| Upregulated in class | na\_neg |
| GeneSet | HALLMARK\_MITOTIC\_SPINDLE |
| Enrichment Score (ES) | -0.2398492 |
| Normalized Enrichment Score (NES) | -1.1210518 |
| Nominal p-value | 0.29642445 |
| FDR q-value | 0.5429728 |
| FWER p-Value | 1.0 |
Table: GSEA Results Summary

  

Fig 1: Enrichment plot: HALLMARK\_MITOTIC\_SPINDLE      
 Profile of the Running ES Score & Positions of GeneSet Members on the Rank Ordered List

  

| PROBE | GENE SYMBOL | GENE\_TITLE | RANK IN GENE LIST | RANK METRIC SCORE | RUNNING ES | CORE ENRICHMENT || 1 | ARHGAP29 |  |  | 54 | 0.863 | 0.0112 | No |
| 2 | FLNB |  |  | 120 | 0.709 | 0.0146 | No |
| 3 | EZR |  |  | 148 | 0.667 | 0.0274 | No |
| 4 | CDC42EP2 |  |  | 235 | 0.576 | 0.0209 | No |
| 5 | MYH9 |  |  | 312 | 0.534 | 0.0159 | No |
| 6 | CDC42EP1 |  |  | 318 | 0.529 | 0.0307 | No |
| 7 | SORBS2 |  |  | 437 | 0.476 | 0.0121 | No |
| 8 | AURKA |  |  | 572 | 0.432 | -0.0122 | No |
| 9 | BIN1 |  |  | 836 | 0.371 | -0.0746 | No |
| 10 | VCL |  |  | 935 | 0.355 | -0.0912 | No |
| 11 | CCNB2 |  |  | 967 | 0.350 | -0.0892 | No |
| 12 | MID1IP1 |  |  | 991 | 0.348 | -0.0850 | No |
| 13 | PDLIM5 |  |  | 1062 | 0.335 | -0.0944 | No |
| 14 | ARHGDIA |  |  | 1153 | 0.323 | -0.1098 | No |
| 15 | UXT |  |  | 1188 | 0.317 | -0.1096 | No |
| 16 | CDC27 |  |  | 1259 | 0.308 | -0.1198 | No |
| 17 | ARHGAP10 |  |  | 1296 | 0.304 | -0.1206 | No |
| 18 | NEK2 |  |  | 1307 | 0.302 | -0.1142 | No |
| 19 | BCAR1 |  |  | 1319 | 0.301 | -0.1081 | No |
| 20 | EPB41L2 |  |  | 1343 | 0.299 | -0.1054 | No |
| 21 | CEP250 |  |  | 1391 | 0.292 | -0.1097 | No |
| 22 | BCR |  |  | 1468 | 0.283 | -0.1223 | No |
| 23 | PLK1 |  |  | 1665 | 0.263 | -0.1692 | No |
| 24 | BIRC5 |  |  | 1667 | 0.263 | -0.1615 | No |
| 25 | FLNA |  |  | 1737 | 0.257 | -0.1730 | No |
| 26 | NCK2 |  |  | 1865 | -0.260 | -0.2006 | No |
| 27 | MID1 |  |  | 1920 | -0.267 | -0.2076 | No |
| 28 | KLC1 |  |  | 1933 | -0.269 | -0.2028 | No |
| 29 | KIF22 |  |  | 2014 | -0.281 | -0.2166 | No |
| 30 | DLG1 |  |  | 2065 | -0.290 | -0.2218 | No |
| 31 | KIF11 |  |  | 2121 | -0.301 | -0.2280 | No |
| 32 | PKD2 |  |  | 2135 | -0.303 | -0.2224 | No |
| 33 | NCK1 |  |  | 2174 | -0.309 | -0.2236 | No |
| 34 | PLEKHG2 |  |  | 2176 | -0.310 | -0.2144 | No |
| 35 | CLIP2 |  |  | 2225 | -0.318 | -0.2181 | No |
| 36 | RABGAP1 |  |  | 2285 | -0.330 | -0.2246 | No |
| 37 | APC |  |  | 2301 | -0.333 | -0.2186 | No |
| 38 | TIAM1 |  |  | 2303 | -0.334 | -0.2087 | No |
| 39 | HOOK3 |  |  | 2415 | -0.356 | -0.2290 | Yes |
| 40 | SAC3D1 |  |  | 2435 | -0.363 | -0.2232 | Yes |
| 41 | KIF1B |  |  | 2443 | -0.365 | -0.2140 | Yes |
| 42 | CENPJ |  |  | 2444 | -0.365 | -0.2029 | Yes |
| 43 | CEP192 |  |  | 2458 | -0.368 | -0.1953 | Yes |
| 44 | KNTC1 |  |  | 2558 | -0.389 | -0.2111 | Yes |
| 45 | DST |  |  | 2614 | -0.404 | -0.2142 | Yes |
| 46 | SMC4 |  |  | 2634 | -0.408 | -0.2071 | Yes |
| 47 | PCM1 |  |  | 2654 | -0.413 | -0.1998 | Yes |
| 48 | RFC1 |  |  | 2690 | -0.421 | -0.1968 | Yes |
| 49 | PREX1 |  |  | 2727 | -0.432 | -0.1937 | Yes |
| 50 | ARHGEF12 |  |  | 2731 | -0.434 | -0.1813 | Yes |
| 51 | CENPF |  |  | 2842 | -0.472 | -0.1977 | Yes |
| 52 | KIF15 |  |  | 2855 | -0.478 | -0.1864 | Yes |
| 53 | NIN |  |  | 2891 | -0.491 | -0.1813 | Yes |
| 54 | BRCA2 |  |  | 2916 | -0.500 | -0.1727 | Yes |
| 55 | STK38L |  |  | 3035 | -0.556 | -0.1888 | Yes |
| 56 | GSN |  |  | 3041 | -0.559 | -0.1731 | Yes |
| 57 | SMC1A |  |  | 3067 | -0.571 | -0.1627 | Yes |
| 58 | KIFAP3 |  |  | 3077 | -0.577 | -0.1476 | Yes |
| 59 | NUMA1 |  |  | 3136 | -0.611 | -0.1452 | Yes |
| 60 | MYO9B |  |  | 3159 | -0.627 | -0.1323 | Yes |
| 61 | SPTBN1 |  |  | 3197 | -0.661 | -0.1224 | Yes |
| 62 | NUSAP1 |  |  | 3199 | -0.663 | -0.1025 | Yes |
| 63 | CDK5RAP2 |  |  | 3205 | -0.672 | -0.0834 | Yes |
| 64 | LMNB1 |  |  | 3246 | -0.711 | -0.0729 | Yes |
| 65 | FARP1 |  |  | 3275 | -0.734 | -0.0583 | Yes |
| 66 | CDK1 |  |  | 3396 | -0.883 | -0.0650 | Yes |
| 67 | FBXO5 |  |  | 3413 | -0.919 | -0.0414 | Yes |
| 68 | CCDC88A |  |  | 3421 | -0.944 | -0.0146 | Yes |
| 69 | TOP2A |  |  | 3457 | -1.020 | 0.0067 | Yes |
| 70 | FGD4 |  |  | 3563 | -1.431 | 0.0210 | Yes |
Table: GSEA details [plain text format]

  

Fig 2: HALLMARK\_MITOTIC\_SPINDLE: Random ES distribution      
 Gene set null distribution of ES for **HALLMARK\_MITOTIC\_SPINDLE**
